# Assessment of Knowledge, Attitude, and Practice Toward Tuberculosis: A Cross‐Sectional Study in Balkh, Afghanistan

**DOI:** 10.1002/hsr2.71338

**Published:** 2025-10-06

**Authors:** Mohammad Masudi, Abdul Wahid Hamidi, Ali Rahimi, Nasar Ahmad Shayan

**Affiliations:** ^1^ Department of Curative Medicine, Faculty of Medicine Jami University Herat Afghanistan; ^2^ Department of Pediatrics, Faculty of Medicine Herat University Herat Afghanistan; ^3^ Department of Curative Medicine, Faculty of Medicine Herat University Herat Afghanistan; ^4^ Department of Epidemiology and Biostatistics Western University London Ontario Canada

**Keywords:** Afghanistan, attitudes, cross‐sectional study, knowledge, outpatients, practice

## Abstract

**Background and Aims:**

Tuberculosis (TB) remains a major public health challenge in Afghanistan, requiring enhanced community engagement for effective control. This study assessed the knowledge, attitudes, and practices (KAP) related to TB among outpatients in Balkh to inform targeted interventions.

**Methods:**

A convenience‐based, face‐to‐face cross‐sectional study was conducted from June 2024 to April 2025 on 867 hospital outpatients in Balkh using a structured questionnaire developed from peer‐reviewed articles. A pilot study with 30 participants showed Cronbach's *α* = 0.767. Descriptive statistics, *χ* ^2^ test, multivariable logistic regression analysis, and Spearman's correlation were performed using SPSS v.27, with statistical significance set at *p* < 0.05.

**Results:**

Of the 867 participants, 63.7%, 52.7%, and 51.4% showed good knowledge, attitude, and practice, respectively. Additionally, good TB‐related knowledge was significantly associated with being married (OR = 6.67), university education (OR = 3.31), prior awareness of TB (OR = 2.29), history of TB treatment (OR = 2.79), and TB vaccination (OR = 1.97) (all *p* < 0.05). Positive attitudes were linked to being married, unskilled employment (OR = 1.83), higher income (OR = 2.50), prior TB awareness (OR = 1.69), and having a window at home (OR = 8.03). Better practice was associated with female gender (OR = 4.20), higher income (OR = 2.02), TB awareness (OR = 1.48), and windowed housing (OR = 6.48), though unvaccinated individuals showed slightly better practice (OR = 1.44). Spearman's correlations showed significant positive associations between KAP scores (all *p* < 0.001).

**Conclusion:**

Significant gaps in TB KAP in Balkh reflect socioeconomic and systemic barriers. Targeted education and community‐based interventions are essential for effective TB control.

## 1 Introduction

Tuberculosis (TB) remains one of the top 10 causes of death worldwide, despite being preventable and curable. According to the World Health Organization (WHO) Global Tuberculosis Report 2023, an estimated 10.6 million people developed TB in 2022, and 1.3 million died, making TB the leading killer among infectious diseases after COVID‐19. The burden is especially high in low‐ and middle‐income countries, where health systems are often strained. TB is further complicated by the emergence of multidrug‐resistant TB (MDR‐TB), which is present in nearly 500,000 new cases annually [1].

Afghanistan is among the 30 high‐burden TB countries and continues to face a major public health crisis due to conflict, displacement, and a fragile health system. The national TB incidence is 180 cases/100,000 population, with significant underreporting, treatment delays, and limited access to diagnostic and care services [2]. Understanding the public's knowledge, attitudes, and practices (KAP) toward TB is essential for tailoring community‐specific interventions and improving control strategies.

Globally, KAP studies serve as powerful tools in identifying misconceptions, stigma, and health‐seeking behaviors associated with TB. In India, a 2021 study found only 15.50% of subjects appeared to have adequate knowledge, 87.33% of subjects had a fair attitude, and around 58% of subjects were reported to have good practices toward TB [3].

In Pakistan, over 90% of participants agreed that TB is dangerous for the community and a majority opted against stigmatizing TB patients (79.1%). People who could read and write were 3.5 times more likely to have a good attitude toward TB compared to those who could not [4]. Similarly, a study in Bangladesh found only 31.8% recognized proximity to a patient as a risk for TB, while 45.5% incorrectly cited saliva via shared cups as the main transmission route. Misconceptions included smoking/drinking (20.8%), heredity (37.5%), and other incorrect sources like uncooked food or sexual contact (8.8%) [5].

Among healthcare workers, TB KAP is equally important. Nurses in a tertiary hospital in Hyderabad, India demonstrated good knowledge but lacked training on infection prevention protocols like mask use [6]. In Nigeria, overall, 60% had good TB knowledge, 50% showed positive attitudes, and 65.2% practiced prevention. Key predictors of good KAP included housing type, education, age, and income source [7].

Several validated tools exist for measuring TB KAP, such as the STBP‐KAPQ developed in China for student patients [8], and the Libyan TB‐KAP instrument for nurses [9]. These tools improve consistency in TB KAP assessments across populations.

Despite global advances in TB research, Afghanistan remains underrepresented in high‐quality KAP studies. However, a few region‐specific studies provide valuable insights. Recent KAP studies in Afghanistan show marked regional variation. In Kabul, 87.7% of hospital outpatients had good knowledge and 96.5% showed positive attitudes, though practical behaviors lagged [10]. In Urozgan, knowledge was poor—only 49.4% recognized airborne transmission, while misconceptions like hereditary spread (51.8%) were common; negative attitudes (55.3%) and poor practices (e.g., only 35.3% screened) were strongly linked to illiteracy and poverty [2]. In Herat, just over half of participants demonstrated good knowledge (61.8%), attitude (50.2%), and practice (51.2%); female gender, higher education, better housing, and higher income were significant predictors of better KAP [11].

Together, these regional findings suggest that while urban areas like Kabul may show promising KAP outcomes, provinces such as Kabul, Urozgan, and Herat continue to struggle with misinformation, stigma, and poor access to TB services. However, Balkh province, a culturally diverse and strategic region in northern Afghanistan, remains poorly studied. This gap in data poses a significant obstacle to formulating effective public health strategies.

The present study aims to assess the KAP related to TB among adults in Balkh. This study will contribute to the national evidence base and support targeted interventions aligned with WHO's End TB Strategy.

## 2 Methods

### 2.1 Study Setting and Design

This convenience‐based cross‐sectional descriptive study was conducted in Balkh, focusing on residents who visited adult outpatient departments (OPD) in public health facility for various medical services. Data collection took place from June 2024 to April 2025 at the Balkh Regional Hospital.

### 2.2 Sample Size and Sampling Type

All individuals meeting the inclusion criteria were invited to participate. The required sample size was calculated using Epi Info software, based on Balkh's estimated population of 2.0 million, with a 95% confidence level, 50% expected prevalence, 5% margin of error, and a design effect of 1.0, yielding a minimum sample size of 385. Ultimately, 867 questionnaires were collected. Participants were recruited using a nonprobability convenience sampling method. The theoretical precision (± 5% at 95% CI) assumed probability sampling; however, as convenience sampling was applied, precision estimates are nominal and generalizability may be limited due to potential selection bias.

### 2.3 Inclusion and Exclusion Criteria

This study included individuals aged ≥ 18 years who had been referred to OPD for medical consultation, as well as those who accompanied patients. Patients younger than 18 years of age and those without outpatient referral were excluded from the study.

### 2.4 Instruments

The questionnaire was adapted from similar studies conducted in Kabul, Afghanistan [2, 10], Ethiopia, Gambia [12, 13], and other countries [14, 15], with modifications made to align better with the local context. The questionnaire was translated into Persian and then back‐translated into English to ensure accuracy. Additionally, the content validity of the instrument was confirmed by ten research experts. The survey was conducted through face‐to‐face interviews, and responses were systematically recorded on paper. A pilot study with 30 participants confirmed the reliability of KAP (Cronbach's *α* = 0.767).

### 2.5 Sociodemographic Questions

This study collected basic sociodemographic information from participants, including age, marital status, occupation, educational level, economic status, and number of family members. Additionally, factors such as tobacco use and living conditions, including the presence of a window at home, were assessed. Furthermore, the participants' history and experience with TB were examined, including whether they had ever been infected, received treatment, had a family member affected by TB, or had been vaccinated against the disease.

### 2.6 Knowledge, Attitude, and Practices Questions

Participants' knowledge of TB was assessed across three domains: transmission, prevention, and symptoms. A binary scoring system was applied, with correct responses assigned a score of 1 and incorrect responses scored as 0, resulting in total knowledge scores ranging from 0 to 12. Based on the median score (7) as in previous study [11], the knowledge levels were classified as poor (0–6) or good (7–12).

Participants' attitudes toward TB were evaluated using items addressing the perceived seriousness of the disease, its transmission, accessibility of treatment, and social stigma. A 5‐point Likert scale was used for scoring. For positively worded statements—such as “TB is a preventable disease,” “TB can be transmitted from one person to another,” “TB is a serious disease,” and “TB treatment is free of cost in Afghanistan”—the responses were scored as follows.

*Strongly Disagree* = 1, *Disagree* = 2, *No Idea* = 3, *Agree* = 4, *Strongly Agree* = 5. Conversely, for negatively phrased statements—such as “TB is perceived as a stigma,” “TB cannot be cured,” “If I get TB infection, I feel ashamed,” “I am afraid of being infected with TB,” and “If I get TB, I feel hopeless”—reverse scoring was applied as follows: *Strongly Agree* = 1, *Agree* = 2, *No Idea* = 3, *Disagree* = 4, *Strongly Disagree* = 5. The cumulative attitude score ranged from 9 to 45, and, based on the median (34), attitudes were categorized as poor (≤ 33) or good (≥ 34).

The participants' practices related to TB were assessed using 11 items. A 5‐point Likert scale was used for each response. For favorable practices—such as “I open room windows for ventilation,” “When traveling, I open the car window for better ventilation,” “If I have TB, I will go to specific hospitals,” “If I have TB, I eat nutritious food,” “If I have TB, I cover my nose and mouth while coughing or sneezing,” and “If I have contact with a TB patient, I wear masks”—responses were scored as follows: *Never* = 1, *Rarely* = 2, *Sometimes* = 3, *Mostly* = 4, *Always* = 5. For items reflecting unfavorable practices—such as “If I have signs and symptoms of TB, I will seek herbal remedies,” “If I have TB, I will treat it with home remedies,” “If I have TB, I intentionally cough and sneeze among people,” “If I have TB, I will go to the clergy seeking treatment,” and “For TB treatment, I use antibiotics without a doctor's consultation”—reverse scoring was applied as follows: *Never* = 5, *Rarely* = 4, *Sometimes* = 3, *Mostly* = 2, *Always* = 1. The total practice score ranged from 11 to 55. Based on the median score (46), the practices were categorized as poor (≤ 45) or good (≥ 46).

### 2.7 Statistical Analysis

Data were analyzed using IBM SPSS Statistics Version 27. Descriptive statistics, including frequencies and percentages, were used to summarize participant characteristics and KAP variables. Associations between sociodemographic factors and KAP outcomes were assessed using Pearson's *χ* ^2^ test; Fisher's exact test was applied when expected cell counts were < 5. Effect sizes for *χ* ^2^ tests were calculated using Cramér's *V* (small = 0.1, medium = 0.3, large = 0.5). Spearman's rank correlation coefficient (*ρ*) was used to assess the relationships between continuous KAP scores. Multivariable binary logistic regression analyses for each KAP outcome were conducted using the Enter method, including all variables with *p* < 0.20 from univariate analyses. Results were reported as odds ratios (OR) with 95% confidence intervals (CI). Model fit was evaluated using the Hosmer–Lemeshow goodness‐of‐fit test. All tests were two‐sided, and *p* < 0.05 was considered statistically significant. This study followed the Strengthening the Reporting of Observational Studies in Epidemiology (STROBE) guidelines for cross‐sectional studies.

## 3 Results

The study included 867 hospital outpatients, of whom 81.5% were male (*n* = 707) and 18.5% were female (*n* = 160). The majority of participants were young adults, with 55.2% aged 18–24 years, followed by 22.8% aged 25–30 years, and 21.9% aged 31 years or older. Most participants were single (63.0%), while 37.0% were married. Regarding occupation, 60.2% reported being in the “other” category (e.g., students, farmers, self‐employed), and 39.8% were classified as unskilled workers.

In terms of educational attainment, 48.0% had completed university‐level education, 20.4% had attained school‐level education, and 31.6% were informal learners or illiterate. Monthly household income varied, with 25.8% earning ≤ 5000 AFN, 42.0% earning between 6000 and 10,000 AFN, and 32.2% earning ≥ 11,000 AFN. Most households were medium‐sized; 48.8% had 6–8 family members, while 28.0% had ≤ 5 members and 23.2% had ≥ 9 members.

Awareness of TB was relatively high, with 73.4% reporting they had heard about TB. A small proportion of participants (6.5%) reported ever having TB themselves, and 7.7% had received TB treatment in the past. Additionally, 20.4% had a family member who had previously contracted TB. Regarding immunization, 26.3% reported having received a TB vaccine. Lastly, the vast majority of participants (94.8%) reported living in homes with windows, which is relevant to ventilation‐related TB prevention measures (Table 1).

**Table 1:** Sociodemographic characteristics (*N* = 867).

Variable	Category	*N* (%)
Sex	Male	707 (81.5)
	Female	160 (18.5)
Age group	18–24	479 (55.2)
	25–30	198 (22.8)
	≥ 31	190 (21.9)
Marital status	Currently single	546 (63.0)
	Married	321 (37.0)
Occupation	Others	522 (60.2)
	Unskilled workers	345 (39.8)
Education	Informal/illiterate	274 (31.6)
	School level	177 (20.4)
	University level	416 (48.0)
Monthly income (AFN)	≤ 5000	224 (25.8)
	6000–10,000	364 (42.0)
	≥ 11,000	279 (32.2)
Family members	≤ 5	243 (28.0)
	6–8	423 (48.8)
	≥ 9	201 (23.2)
Heard about TB	Yes	636 (73.4)
	No	231 (26.6)
Ever had TB	Yes	56 (6.5)
	No	811 (93.5)
Ever received TB treatment	Yes	67 (7.7)
	No	800 (92.3)
Family member had TB	Yes	177 (20.4)
	No	690 (79.6)
Received vaccine	Yes	228 (26.3)
	No	639 (73.7)
Home has window	Yes	822 (94.8)
	No	45 (5.2)

**Figure 1:**
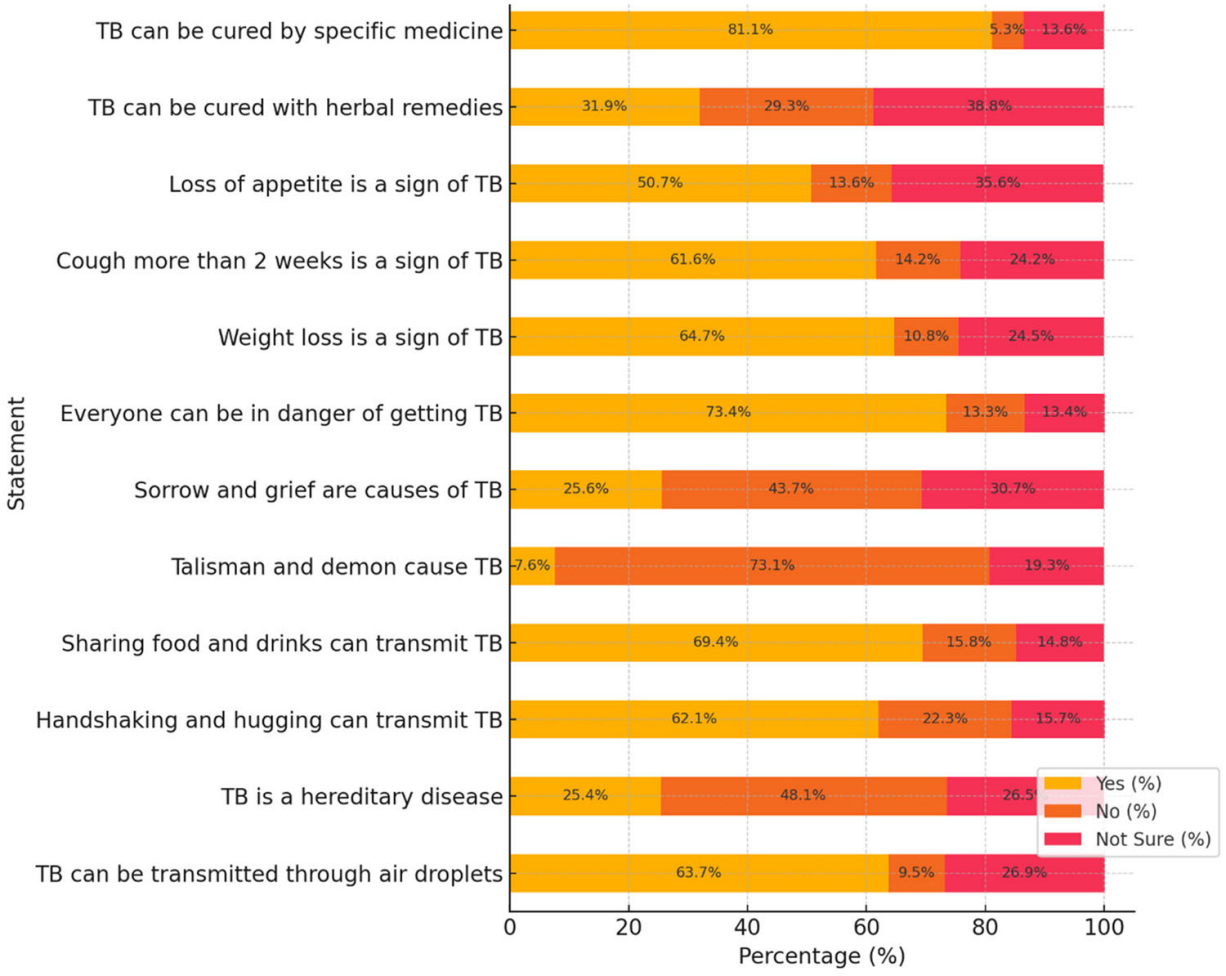
Knowledge of TB among hospital outpatients in Balkh, Afghanistan (*N* = 867).

Most participants (69.4%) believed TB could be transmitted through sharing food and drinks, and 63.7% correctly identified transmission through air droplets. A large majority (73.1%) rejected the misconception that talismans and demons cause TB, and 73.4% recognized that everyone is at risk of contracting the disease. Regarding symptoms, weight loss was the most commonly identified sign (64.7%), and importantly, 81.1% of respondents correctly stated that TB can be cured with specific medicine (Figure 1).

**Figure 2:**
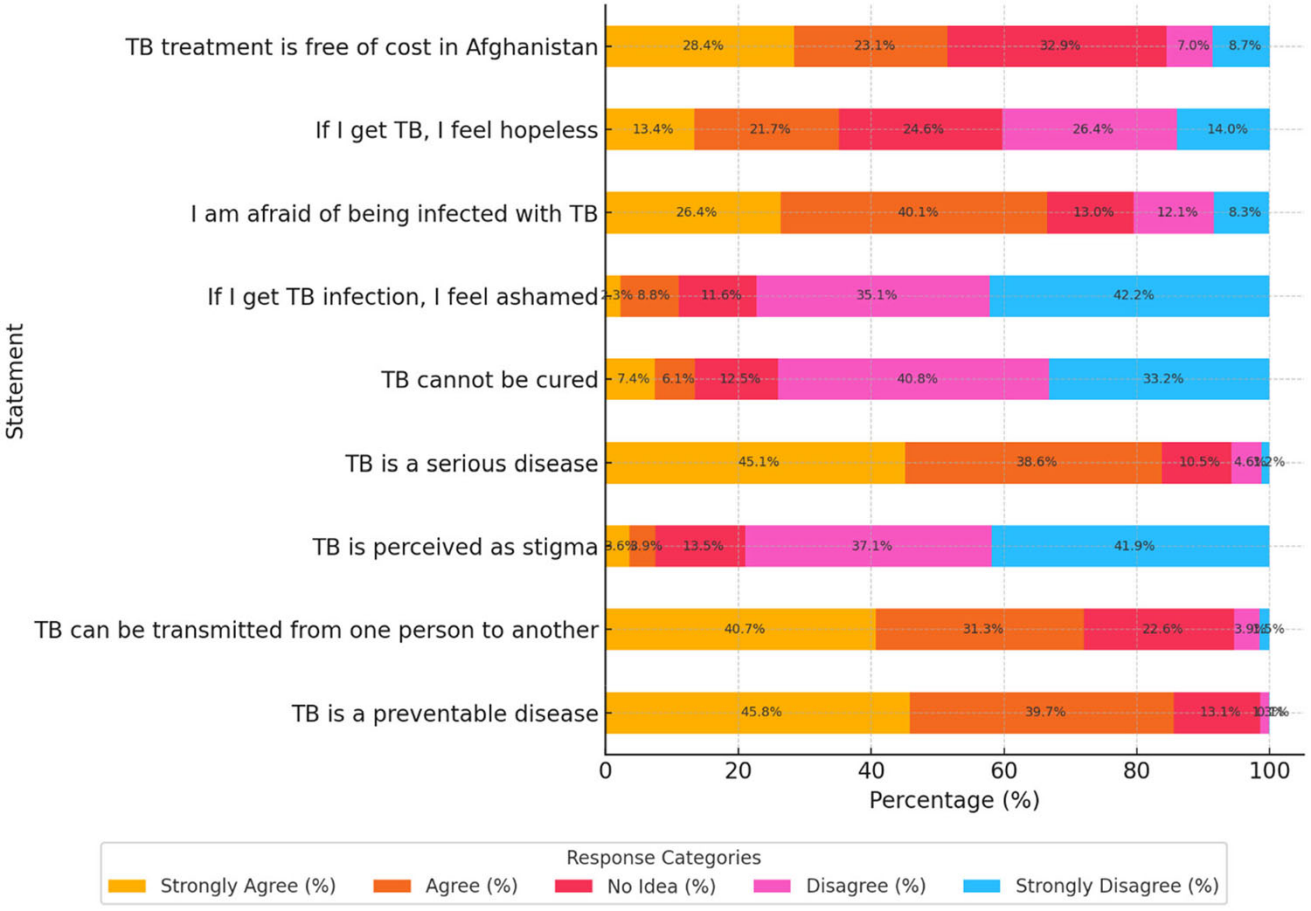
Attitude of TB among hospital outpatients in Balkh, Afghanistan (*N* = 867).

A vast majority of respondents agreed that TB is a preventable disease, with 45.8% strongly agreeing and 39.7% agreeing. Similarly, 40.7% strongly agreed and 31.3% agreed that TB can be transmitted from one person to another, indicating high awareness of its infectious nature. The perception of TB as a stigma was low, with 41.9% strongly disagreeing and 37.1% disagreeing with that TB stigmatized, while only 3.6% strongly agreed with the stigma statement. Recognition of TB as a serious illness was high, with 45.1% strongly agreeing and 38.6% agreeing. Most participants disagreed with the misconception that TB cannot be cured; 40.8% disagreed, and 33.2% strongly disagreed, while only 7.4% strongly agreed with this incorrect belief. Regarding emotional reactions to illness, a majority rejected negative self‐perceptions: 42.2% strongly disagreed and 35.1% disagreed that they would feel ashamed if infected with TB, while 26.4% disagreed and 14.0% strongly disagreed that they would feel hopeless. However, fear of infection remained relatively high, with 40.1% agreeing and 26.4% strongly agreeing that they are afraid of being infected. Lastly, the awareness of healthcare support was moderate. Only 28.4% strongly agreed and 23.1% agreed that TB treatment is free of cost in Afghanistan, while 32.9% responded with “no idea,” and a combined 15.7% disagreed or strongly disagreed (Figure 2).

**Figure 3:**
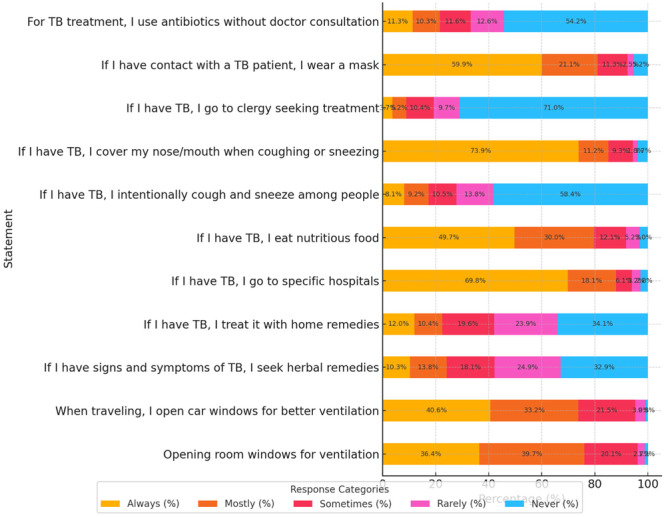
Practice of TB among hospital outpatients in Balkh, Afghanistan (*N* = 867).

A large majority of participants reported consistently opening room windows for ventilation, with 36.4% always and 39.7% mostly doing so. Similarly, 40.6% always and 33.2% mostly opened car windows during travel for better air circulation. Regarding seeking formal care, 69.8% of respondents reported that they would always go to specific hospitals if diagnosed with TB, while only 2.8% stated they would never do so. Nutritional support was also recognized, with 49.7% always and 30.0% mostly reporting they would eat nutritious food during TB illness. In terms of respiratory hygiene, a significant proportion stated they would always cover their nose or mouth when coughing or sneezing if they had TB (73.9%), while only 3.7% never practiced this behavior. Conversely, 58.4% of participants stated they would never intentionally cough or sneeze among people, though a minority (8.1%) admitted they would always do so. The use of masks after TB exposure was relatively high, with 59.9% always and 21.1% mostly wearing masks if they had contact with a TB patient. However, some participants endorsed inappropriate practices. A notable proportion expressed reliance on traditional remedies: 10.3% would always seek herbal treatments for TB symptoms, and 12.0% would always treat TB with home remedies. Religious‐based treatment‐seeking was rare, with 3.7% stating they would always go to clergy, whereas 71.0% reported they would never do so. Importantly, over half of the participants (54.2%) reported they would never use antibiotics without doctor consultation, while 11.3% admitted they would always self‐medicate with antibiotics if they had TB (Figure 3).

*In respect of knowledge*, a higher proportion of males (50.6%) had good knowledge compared to females (13.0%) (*p* = 0.043). Single participants had better knowledge (35.3%) than married individuals (28.4%) (*p* < 0.001). University‐level participants reported the highest good knowledge (33.8%), followed by informal/illiterate individuals (17.6%) and school level (12.2%) (*p* < 0.001). Only 5.9% of participants with a history of TB treatment had good knowledge compared to 57.8% of those without (*p* = 0.027). Participants with a family history of TB (14.6%) had better knowledge than those without (49.0%) (*p* = 0.012). Those who had received a TB vaccine (19.7%) were more likely to have good knowledge than those who had not (43.9%) (*p* < 0.001). Living in a home with windows (61.4%) was associated with higher knowledge compared to those without windows (2.3%) (*p* = 0.006). Participants who had heard about TB (51.3%) had better knowledge than those who had not (12.3%) (*p* < 0.001). Regarding *Attitude*, single participants had a higher proportion of good attitude (31.4%) compared to married individuals (21.3%) (*p* = 0.026). Monthly income was significantly related to attitude, with those earning ≤ 5000 AFN having a lower good attitude (11.3%) compared to those earning 6000–10,000 AFN (20.3%) and ≥ 11,000 AFN (21.1%) (*p* < 0.001). Participants who had heard about TB (41.5%) were more likely to have a good attitude than those who had not (11.2%) (*p* < 0.001). Living in a home with windows (52.0%) was associated with a better attitude compared to those without windows (0.7%) (*p* < 0.001). Practically, males had a higher proportion of good practice (34.6%) compared to females (14.0%) (*p* < 0.001). Single participants (28.1%) had better practice than married individuals (20.4%) (*p* = 0.003). Those in the “other” occupation category (33.0%) had better practice than unskilled workers (15.6%) (*p* < 0.001). University graduates (24.7%) reported higher good practice than informal/illiterate participants (13.4%) and school‐level participants (10.5%) (*p* = 0.045). Participants earning ≤ 5000 AFN (11.0%) had lower good practice than those earning 6000–10,000 AFN (20.3%) and ≥ 11,000 AFN (17.3%) (*p* = 0.040). Those with ≤ 5 family members (15.3%) had better practice than those with ≥ 9 members (11.5%) (*p* = 0.035). Participants without a family TB history had higher good practice (36.8%) than those with such a history (11.8%) (*p* = 0.007). Participants without prior TB treatment had higher good practice (43.8%) than those with treatment history (4.7%) (*p* = 0.031). Participants who had heard about TB (38.4%) had higher good practice than those who had not (10.1%) (*p* < 0.001), and living in a home with windows (47.8%) was associated with better practice than without windows (0.8%) (*p* < 0.001) (Table 2).

**Table 2:** Univariate analysis of TB knowledge, attitude, and practice among hospital outpatients in Balkh, Afghanistan (*N* = 867).

Variable	Category	Good knowledge	*p*	Good attitude	*p*	Good practice	*p*
Sex	Male	439 (50.6)	0.043*	365 (42.1)	0.179	300 (34.6)	< 0.001*
	Female	113 (13.0)		92 (10.6)		121 (14.0)	
Age group	18–24	288 (33.2)	0.053	251 (29.0)	0.118	219 (25.3)	0.175
	25–30	136 (15.7)		95 (11.0)		104 (12.0)	
	≥ 31	128 (14.8)		111 (12.8)		98 (11.3)	
Marital status	Currently single	306 (35.3)	< 0.001*	272 (31.4)	0.026*	244 (28.1)	0.003*
	Married	246 (28.4)		185 (21.3)		177 (20.4)	
Occupation	Others	344 (39.7)	0.093	262 (30.2)	0.068	286 (33.0)	< 0.001*
	Unskilled workers	208 (24.0)		195 (22.5)		135 (15.6)	
Education	Informal/illiterate	153 (17.6)	< 0.001*	140 (16.1)	0.777	116 (13.4)	0.045*
	School level	106 (12.2)		93 (10.7)		91 (10.5)	
	University level	293 (33.8)		224 (25.8)		214 (24.7)	
Monthly income	≤ 5000	129 (14.9)	0.060	98 (11.3)	< 0.001*	95 (11.0)	0.040*
	6000–10,000	234 (27.0)		176 (20.3)		176 (20.3)	
	≥ 11,000	189 (21.8)		183 (21.1)		150 (17.3)	
Family members	≤ 5	164 (18.9)	0.299	129 (14.9)	0.650	133 (15.3)	0.035*
	6–8	266 (30.7)		217 (25.0)		188 (21.7)	
	≥ 9	122 (14.1)		111 (12.8)		100 (11.5)	
Heard about TB	Yes	445 (51.3)	< 0.001*	360 (41.5)	< 0.001*	333 (38.4)	< 0.001*
	No	107 (12.3)		97 (11.2)		88 (10.1)	
Had TB	Yes	36 (4.2)	0.921	25 (2.9)	0.211	33 (3.8)	0.108
	No	516 (59.5)		432 (49.8)		388 (44.8)	
Received TB treatment	Yes	51 (5.9)	0.027*	38 (4.4)	0.494	41 (4.7)	0.031*
	No	501 (57.8)		419 (48.3)		380 (43.8)	
Family TB history	Yes	127 (14.6)	0.012*	98 (11.3)	0.427	102 (11.8)	0.007*
	No	425 (49.0)		359 (41.4)		319 (36.8)	
Received vaccine	Yes	171 (19.7)	< 0.001*	126 (14.5)	0.369	99 (11.4)	0.071
	No	381 (43.9)		331 (38.2)		322 (37.1)	
Home has window	Yes	532 (61.4)	0.006*	451 (52.0)	< 0.001*	414 (47.8)	
	No	20 (2.3)		6 (0.7)		7 (0.8)	

*Note:* *Indicates statistical significance at *p*≤0.05.

In the adjusted analysis for TB‐related knowledge, participants who were married had significantly higher odds of good knowledge compared to those who were single (OR = 6.67, 95% CI: 3.98–11.11) (*p* < 0.001). Likewise, those with university‐level education had higher odds of good knowledge compared to individuals with informal education or no literacy (OR = 3.31, 95% CI: 2.04–5.38) (*p* < 0.001). Participants who had heard about TB were more likely to have good knowledge than those who had not (OR = 2.29, 95% CI: 1.59–3.31) (*p* < 0.001), and those who had received TB treatment also showed higher odds compared to those who had not (OR = 2.79, 95% CI: 1.10–7.08) (*p* = 0.031). In addition, individuals who had received the TB vaccine were more likely to have good knowledge than those unvaccinated (OR = 1.97, 95% CI: 1.35–2.86) (*p* < 0.001). Regarding attitude, unskilled workers had significantly higher odds of good attitude compared to participants with other occupations (e.g., students, farmers) (OR = 1.83, 95% CI: 1.28–2.65) (*p* = 0.001). Similarly, those earning ≥ 11,000 AFN/month had better attitude compared to those with monthly income ≤ 5000 AFN (OR = 2.50, 95% CI: 1.64–3.79) (*p* < 0.001). Participants who had heard about TB had higher odds of good attitude than those who had not (OR = 1.69, 95% CI: 1.18–2.42) (*p* = 0.004), and living in a home with a window was strongly associated with higher odds of good attitude than homes without windows (OR = 8.03, 95% CI: 3.16–20.38) (*p* < 0.001). Married individuals also had better attitudes compared to single individuals (OR = 1.61, 95% CI: 1.05–2.44) (*p* = 0.026). For TB‐related practice, females had significantly higher odds of good practice than males (OR = 4.20, 95% CI: 2.69–6.54) (*p* < 0.001), and participants earning ≥ 11,000 AFN had better practice than those earning ≤ 5000 AFN (OR = 2.02, 95% CI: 1.32–3.11) (*p* = 0.001). Those who had heard about TB showed higher odds of appropriate practice than those who had not (OR = 1.48, 95% CI: 1.02–2.13) (*p* = 0.039), and living in a home with a window was associated with substantially better practice than homes without windows (OR = 6.48, 95% CI: 2.46–17.07) (*p* < 0.001). Interestingly, unvaccinated individuals had slightly higher odds of good practice compared to vaccinated individuals (OR = 1.44, 95% CI: 1.01–2.05) (*p* = 0.041) (Table 3).

**Table 3:** Multivariable logistic regression analysis of TB knowledge, attitude, and practice among hospital outpatients in Balkh, Afghanistan (*N* = 867).

Variables		*p*	OR	95% CI for OR		*p*	OR	95% CI for OR		*p*	OR	95% CI for OR	
				Lower	Upper			Lower	Upper			Lower	Upper
Sex	Male	0.104	0.686	0.436	1.080	0.003	0.532	0.353	0.801	< 0.001*	0.238	0.153	0.372
	Female (Ref)												
Age group	18–24	0.122	1.554	0.889	2.716	0.616	1.134	0.693	1.856	0.747	0.919	0.550	1.536
	25–30	0.177	1.439	0.849	2.441	0.288	0.779	0.491	1.235	0.647	1.118	0.694	1.801
	≥ 31 (Ref)												
Marital status	Currently single	< 0.001*	0.150	0.090	0.251	0.026	0.622	0.409	0.946	0.062	0.663	0.431	1.021
	Currently married (Ref)												
Occupation	Others	0.952	0.988	0.672	1.453	0.001	0.545	0.378	0.784	0.053	1.443	0.995	2.094
	Unskilled workers (Ref)												
Education	Informal education and illiterate	< 0.001*	0.302	0.186	0.491	0.165	0.739	0.482	1.132	0.096	0.686	0.440	1.069
	School level	0.323	0.806	0.525	1.236	0.606	1.115	0.738	1.684	0.138	1.380	0.902	2.111
	University level (Ref)												
Monthly income	≤ 5000	0.111	0.699	0.450	1.086	< 0.001*	0.400	0.264	0.607	0.001	0.494	0.322	0.757
	6000–10,000	0.846	0.963	0.659	1.408	< 0.001*	0.489	0.340	0.704	0.624	0.913	0.634	1.314
	≥ 11,000 (Ref)												
Family members	≤ 5	0.229	1.329	0.836	2.111	0.588	0.890	0.583	1.358	0.588	1.126	0.733	1.729
	6–8	0.217	1.282	0.864	1.902	0.786	0.949	0.652	1.383	0.156	0.760	0.520	1.111
	≥ 9 (Ref)												
Heard about TB	Yes	< 0.001*	2.294	1.591	3.309	0.004	1.688	1.177	2.422	0.039	1.476	1.020	2.134
	No (Ref)												
Had TB	Yes	0.321	0.608	0.228	1.624	0.147	0.495	0.192	1.279	0.830	1.108	0.435	2.824
	No (Ref)												
Received TB treatment	Yes	0.031	2.792	1.101	7.078	0.060	2.256	0.966	5.269	0.174	1.740	0.783	3.866
	No (Ref)												
Family TB history	Yes	0.082	1.465	0.952	2.256	0.789	1.054	0.718	1.547	0.210	1.288	0.867	1.914
	No (Ref)												
Received vaccine	Yes	< 0.001*	1.965	1.348	2.863	0.792	1.047	0.746	1.469	0.041	0.693	0.488	0.986
	No (Ref)												
Home has window	Yes	0.584	1.217	0.602	2.457	< 0.001*	8.025	3.160	20.382	< 0.001*	6.479	2.460	17.067
	No (Ref)												
Constant		0.359	1.759			0.079	0.317			0.880	0.903		

*Note:* *Indicates statistical significance at *p*≤0.05.

Spearman's correlation analysis revealed statistically significant positive correlations among KAP scores related to TB. Knowledge score was moderately correlated with attitude score (*r* = 0.291, *p* < 0.001) and practice score (*r* = 0.349, *p* < 0.001). Similarly, attitude score was positively associated with practice score (*r* = 0.273, *p* < 0.001). Knowledge grade showed a strong correlation with knowledge score (*r* = 0.839, *p* < 0.001), and also had weak but significant correlations with attitude score (*r* = 0.299, *p* < 0.001) and practice score (*r* = 0.290, *p* < 0.001). Attitude grade was strongly associated with attitude score (*r* = 0.868, *p* < 0.001), and showed weaker associations with knowledge score (*r* = 0.244, *p* < 0.001), practice score (*r* = 0.258, *p* < 0.001), and knowledge grade (*r* = 0.240, *p* < 0.001). Likewise, practice grade had a strong correlation with practice score (*r* = 0.867, *p* < 0.001), and weaker but significant correlations with knowledge score (*r* = 0.306, *p* < 0.001), attitude score (*r* = 0.264, *p* < 0.001), knowledge grade (*r* = 0.264, *p* < 0.001), and attitude grade (*r* = 0.241, *p* < 0.001) (Table 4).

**Table 4:** Spearman's correlations between knowledge, attitude, and practices toward tuberculosis.

		Knowledge score	Attitude score	Practice score	Knowledge grade	Attitude grade	Practice grade
Knowledge score	Correlation coefficient	1.000					
	*p*						
Attitude score	Correlation coefficient	0.291	1.000				
	*p*	< 0.001*					
Practice score	Correlation coefficient	0.349	0.273	1.000			
	*p*	< 0.001*	< 0.001*				
Knowledge grade	Correlation coefficient	0.839	0.299	0.290	1.000		
	*p*	< 0.001*	< 0.001*	< 0.001*			
Attitude grade	Correlation coefficient	0.244	0.868	0.258	0.240	1.000	
	*p*	< 0.001*	< 0.001*	< 0.001*	< 0.001*		
Practice grade	Correlation coefficient	0.306	0.264	0.867	0.264	0.241	1.000
	*p*	< 0.001*	< 0.001*	< 0.001*	< 0.001*	< 0.001*	

*Note:* *Indicates statistical significance at *p*≤0.05.

## 4 Discussion

This study evaluated the KAP concerning TB among outpatient populations in Balkh Province, northern Afghanistan. Overall, 63.7% of participants demonstrated good knowledge of TB, while 36.3% had poor knowledge. Knowledge levels were significantly higher among males (50.6%), single individuals (35.3%), and those with university education (33.8%) compared to their counterparts (*p* < 0.05). Participants with a history of TB treatment, a family history of TB, or prior TB vaccination also showed significantly better knowledge, as did those living in homes with windows (61.4%) and those who had previously heard about TB (51.3%) (all *p* < 0.05). Attitudinally, 52.7% displayed a positive attitude toward TB, with significantly more favorable attitudes among single individuals, those earning higher incomes, those who had heard about TB (41.5%), and those living in well‐ventilated homes (52.0%) (*p* < 0.001). In terms of practice, 48.6% reported good preventive behaviors. Better TB‐related practices were significantly associated with being female (14.0%), single (28.1%), educated (especially university level), and employed in occupations such as farming or studying (33.0%) (*p* < 0.05). Higher income, fewer family members, a family history of TB, previous treatment, access to TB information, and ventilated housing were also strong predictors of good practice (all *p* < 0.05).

When compared to other regional studies in Afghanistan, the findings from Balkh reflect a *moderate level of knowledge and attitudes*, but *relatively lower practice scores*. In the present study, 63.7% of participants demonstrated good knowledge, which is higher than that reported in *Urozgan (49.4%)* and *Herat (61.8%)*, but notably lower than in *Kabul (87.7%)*. Attitude levels in Balkh (52.7%) were comparable to Herat (50.2%), slightly better than Urozgan (44.7% with positive attitudes), but still below the high attitude levels observed in Kabul (96.5%). However, *practice outcomes in Balkh were the weakest*, with only 48.6% reporting good practices—similar to Herat (51.2%), but lower than Kabul's relatively strong practical engagement and even below Urozgan's limited indicators like cough hygiene (54.1%) [2, 10, 11]. Approximately 73.4% of participants had heard of TB—a figure considerably lower than the rates reported in studies from Ethiopia, Libya, Malaysia, and Iraq, where awareness exceeded 90% [16, 17, 18, 19, 20]. Such disparities may stem from the disruption of health communication due to ongoing political instability, limited educational access, and reduced public health outreach in Afghanistan. Furthermore, only 63.7% correctly identified airborne transmission via droplets, while misconceptions—such as transmission through hugging or food‐sharing—remained widespread. These beliefs were similarly prevalent in Uruzgan, where over half of respondents believed TB was hereditary or food‐borne [21]. In contrast with the significantly better understanding of transmission observed in Kabul (87.7%) and Ethiopia (95.5%) [22, 23]. Recognition of TB symptoms was similarly mixed. While 61.6% correctly identified persistent cough as a cardinal symptom—comparable to Uruzgan (50.6%) and higher than Ethiopia (39.4%) [21, 24] —awareness of systemic symptoms such as chest pain and night sweats was poor. In contrast, over 85% of respondents in the Ethiopian Demographic and Health Survey recognized cough and at least two additional symptoms [23], possibly reflecting long‐term investments in community‐based health education programs. Comparable findings were reported in studies from Ethiopia and Gambia, where participants similarly recognized these key symptoms [24, 25].

While many acknowledge that the disease is treatable. However, a substantial proportion (31.9%) still expressed belief in traditional or herbal remedies, indicating a persistent cultural inclination toward nonbiomedical treatment approaches. Similar patterns have been observed in rural areas of Ethiopia and Uruzgan Province, where traditional healers frequently serve as the first point of contact for individuals with TB symptoms, particularly in contexts with limited access to formal healthcare services [21, 24]. These findings underscore the importance of culturally sensitive interventions that acknowledge local beliefs while promoting evidence‐based treatment. Most participants (84.5%) acknowledged TB as a preventable disease, and 83.7% recognized its contagious nature, indicating moderately favorable attitudes. However, 39.9% denied the existence of stigma, a finding that contrasts with Ethiopian data where stigma manifested through shame, concealment, and fear of rejection [23]. This discrepancy may reflect genuine sociocultural differences or could result from social desirability bias affecting respondents' answers. A notable finding of this study was the statistically significant and strong positive correlation observed between knowledge, attitudes, and practices related to TB (*p* < 0.001). This relationship underscores the interdependent nature of these domains and highlights the value of integrated health education strategies—suggesting that improvement in one area may lead to favorable outcomes in the others. Similar associations have been documented in previous studies conducted in Kabul and Ethiopia, reinforcing the global relevance of comprehensive, behaviorally informed TB control programs [22, 23]. Multivariate analysis indicated that younger age, male gender, and higher income levels were independently associated with higher knowledge and more positive attitudes. These findings align with results from Uruzgan, where socioeconomic status significantly predicted better KAP outcomes [21]. The Ethiopian national survey similarly observed that TB patients and their immediate contacts scored higher in knowledge domains, likely due to their more frequent interaction with health services [23]. Also, higher education was associated with better knowledge of TB; this is in line with a study conducted in Khyber Pakhtunkhwa, Pakistan, where education level was correlated with better knowledge of TB [26]. These findings reaffirm the crucial role of socioeconomic determinants in shaping health‐related behaviors.

It is a well‐known fact that knowledge can influence people's practices regarding prevention [27]. Encouragingly, 73.9% of participants reported covering their mouths when coughing, and 59.9% used face masks around TB patients. These figures are higher than those reported in Ethiopia (48%), Iran (42.6%), and Thailand (55.5%) [24, 28, 29]. This suggests a relatively positive uptake of basic preventive measures. Nevertheless, practices remain suboptimal and likely influenced by regional differences in public health messaging and access to materials such as masks. Awareness of drug‐resistant TB (DR‐TB) was notably limited, and engagement with community‐based healthcare services was minimal. These findings align with studies from Ethiopia, where only 24% of respondents had heard of DR‐TB and health extension workers were underutilized in case detection [23]. Strengthening awareness and integrating community health workers into TB education and case detection remain critical unmet needs. The broader political and structural context in Afghanistan has significantly influenced TB control efforts. Since the return of the Taliban regime, international sanctions and the withdrawal of foreign aid have debilitated the healthcare infrastructure. The Sehatmandi program, once the backbone of national health service delivery, has largely ceased functioning [30]. In this fragile environment, TB patients may remain undiagnosed, and awareness campaigns are nearly absent. Internally displaced populations are particularly vulnerable to TB transmission, further exacerbating the burden. These contextual factors must be considered when interpreting the study's findings, as they fundamentally constrain health‐seeking behavior and access to care.

To bridge these gaps, a coordinated, policy‐driven response is urgently needed, prioritizing culturally adapted health education, expanded community‐based services, and integration of trained community health workers into TB programs, particularly for contact tracing and early detection. In interpreting these findings, it is important to note that the marked male predominance in our sample (81.5%) likely reflects the combined effect of convenience sampling and sociocultural patterns in OPD attendance in Afghanistan, where men more often accompany or seek care at public facilities. This imbalance may have influenced overall KAP estimates, as prior research suggests women in this setting can demonstrate different TB‐related practices, potentially underestimating certain positive behaviors. Future studies should incorporate strategies such as community‐based recruitment, household surveys, and female outreach clinics to ensure more balanced gender representation. Our results also highlight several direct implications for TB control under the National Tuberculosis Programme (NTP). The strong association between living in a home with a window and both positive attitudes (OR = 8.03) and good practices (OR = 6.48) underscores the need for ventilation‐focused health messaging and urban planning that reduces overcrowding. Furthermore, the finding that only half of the participants recognized TB treatment as free in Afghanistan suggests a missed communication opportunity for the NTP/DOTS strategy. Contact tracing, especially through trained community health workers, should be prioritized to reach household members and close contacts of TB patients, given the observed association between TB awareness and better KAP scores. Finally, the significantly higher adjusted odds of good practice among women (OR = 4.20) present an opportunity to develop women‐led outreach and peer‐education programs that can leverage existing social networks to promote TB prevention and care‐seeking behaviors.

## 5 Conclusion

This study reveals persistent gaps in TB‐related knowledge (63.7%), attitudes (52.7%), and practices (48.6%) among outpatient populations in Balkh Province, Afghanistan. Key predictors of better KAP included higher education, prior TB awareness, higher income, and living in well‐ventilated housing, while female participants demonstrated markedly better TB‐related practices. The male‐heavy sample (81.5%), likely due to sociocultural patterns and convenience sampling, may have led to underestimation of certain positive behaviors among women. Addressing these disparities will require targeted, culturally sensitive interventions, enhanced public awareness of free TB services, ventilation‐focused health promotion, and the strategic use of community health workers for contact tracing and outreach. Future research should adopt more representative sampling and explore the effectiveness of women‐led and community‐based approaches to strengthen Afghanistan's alignment with the WHO End TB Strategy.

## Author Contributions

**Mohammad Masudi:** conceptualization, writing – original draft, methodology, writing – review and editing, validation, software, data curation, supervision, formal analysis. **Abdul Wahid Hamidi:** writing – original draft, writing – review and editing, methodology, conceptualization. **Ali Rahimi:** conceptualization, writing – original draft, writing – review and editing, methodology. **Nasar Ahmad Shayan:** writing – original draft, writing – review and editing, supervision, methodology. All authors have read and approved the final version of the manuscript.

## Ethics Statement

The study protocol was reviewed and approved by the Institutional Review Board of Jami University (Approval No. J.2024.1.27.9). All procedures performed in this study were in accordance with the ethical standards of the institutional and/or national research committee and with the 1964 Declaration of Helsinki and its later amendments.

## Consent

Written informed consent was obtained from all participants prior to data collection. Participation was voluntary, and confidentiality of responses was maintained throughout the study. All authors have read and approved the final version of the manuscript and consent to its publication.

## Conflicts of Interest

The authors declare no conflicts of interest.

## Transparency Statement

The lead author Mohammad Masudi affirms that this manuscript is an honest, accurate, and transparent account of the study being reported; that no important aspects of the study have been omitted; and that any discrepancies from the study as planned (and, if relevant, registered) have been explained.

## Data Availability

The data sets generated and/or analyzed during the current study are available from the corresponding author, Dr. Mohammad Masudi (mhmasoudy313@gmail.com), upon reasonable request. All authors had full access to all of the data in this study and take complete responsibility for the integrity of the data and the accuracy of the data analysis.
